# Hospital preparedness during epidemics using simulation: the case of COVID-19

**DOI:** 10.1007/s10100-021-00779-w

**Published:** 2021-09-27

**Authors:** Daniel Garcia-Vicuña, Laida Esparza, Fermin Mallor

**Affiliations:** 1 Institute of Smart Cities, Public University of Navarre, Campus Arrosadia, 31006 Pamplona, Spain; 2 Hospital Compound of Navarre, Irunlarrea, 3, 31008 Pamplona, Spain

**Keywords:** Discrete event simulation model, COVID-19, Bed occupancy, Hospital resources planning, Gompertz growth model

## Abstract

This paper presents a discrete event simulation model to support decision-making for the short-term planning of hospital resource needs, especially Intensive Care Unit (ICU) beds, to cope with outbreaks, such as the COVID-19 pandemic. Given its purpose as a short-term forecasting tool, the simulation model requires an accurate representation of the current system state and high fidelity in mimicking the system dynamics from that state. The two main components of the simulation model are the stochastic modeling of patient admission and patient flow processes. The patient arrival process is modelled using a Gompertz growth model, which enables the representation of the exponential growth caused by the initial spread of the virus, followed by a period of maximum arrival rate and then a decreasing phase until the wave subsides. We conducted an empirical study concluding that the Gompertz model provides a better fit to pandemic-related data (positive cases and hospitalization numbers) and has superior prediction capacity than other sigmoid models based on Richards, Logistic, and Stannard functions. Patient flow modelling considers different pathways and dynamic length of stay estimation in several healthcare stages using patient-level data. We report on the application of the simulation model in two Autonomous Regions of Spain (Navarre and La Rioja) during the two COVID-19 waves experienced in 2020. The simulation model was employed on a daily basis to inform the regional logistic health care planning team, who programmed the ward and ICU beds based on the resulting predictions.

## Introduction

The COVID-19 pandemic presents a major global health threat. Since the outbreak in China in early December 2019, more than 180 million confirmed cases, and close to four million deaths from COVID-19 infection (up to the end of June 2021 https://coronavirus.jhu.edu/map.html) have been recorded. Regularly updated information on the COVID-19 outbreak is also available on the websites of the European Centre for Disease Prevention and Control (ECDC), the European Commission (EC), and the World Health Organization (WHO). This outbreak has brought changes in health care delivery, and in hospital systems stretched by the sudden increase in demand. The treatment of COVID-19 patients requires dedicated resources, material, and personnel. The pandemic has had a particularly intense impact on Intensive Care Units (ICU), which require highly specialized personnel and costly technical apparatus. Accurate planning requires accurate prediction of resource needs. In addition, hospitalized COVID-19 patients need to be isolated from other types of patients, which makes advance preparation of wards necessary. Therefore, the management of both ICU and ward beds for COVID-19 patients benefits from accurate short-term demand forecasting. Other resource needs, such as personnel requirements, can be calculated from bed demand numbers. Usually, the hospitalization bed is still widely used as a hospital (ICU) management parameter both at the strategic and operational levels.

Hospitals are complex systems evolving in a stochastic environment with a level of uncertainty which intensifies during pandemics due to lack of knowledge about the spread of the disease and its consequences for those infected. In this unsettled context, simulation emerges as a suitable tool of analysis, since it is able to reproduce both the complexity of the system and the variability and uncertainty of the environment, as well as being eligible for use in combination with other analytical techniques. The literature contains numerous bibliographical references relating to the use of simulation models for decision making in the healthcare context. Most applications use simulation to support strategic decisions, usually for resource sizing, scheduling, or management. All these cases require the design of a simulation model to reproduce stationary state healthcare system performance and evaluate resource levels, patient flow management policies, and the long-term decision making process. The recommendations obtained from the simulation analysis are intended for a pre-determined implementation period.

However, a simulation model designed to enable tactical decisions for the provision of specialized health resources during the current outbreak has to focus on the transition period, if it is to generate a short-term projection of the current state of the hospital. To achieve this goal, the simulation model needs to account for non-stationary and non-periodic patient input to the hospital, a complex hospital situation at the Simulation Starting Point (SSP), variation in the patient hospital length of stay (LoS) pattern and censored data. This paper presents a Discrete Event Simulation (DES) model combining dynamic forecasting to predict (simulate) new patient arrivals and the reproduction of patient flow patterns and designed to address all the issues just raised. The simulation process yields future resource-use scenarios to inform the health authorities of future needs and give them time to plan. In fact, the results of the simulation model were used on a daily basis during the successive waves of the pandemic (from March 2020 to May 2021) by local governments of two Spanish regions and by the Spanish Ministry of Health as a health resources planning instrument. Therefore, the main feature of the simulation model presented here is its capacity to reproduce the evolution of the health system from its current state, in a non-stationary and changing environment, thus providing a useful forecasting tool.

The main contribution of this paper is our proposal for a new simulation framework enabling short-term (from days to a few weeks) prediction of critical resource needs for the care of COVID-19 patients and our account of its use by health authorities during the COVID-19 pandemic waves. The simulation framework can be adapted for application in potential future outbreaks. To achieve this main contribution our research includes:

- A method to simulate patient arrival times based on Population Growth (PG) models. These are better suited for the prediction of hospitalization (and positive cases) series than other mathematical alternatives such as SIR-type models, which require detailed knowledge of the spread of the disease throughout the population and the estimation of many parameters. PG models produce S-shape curves able to represent the evolution of pandemic variables, such as positive cases and hospitalizations, from beginning to end of the outbreak.
- A statistical analysis of the accuracy of four different PG models in simulating and forecasting the spread of the pandemic.
- The representation of the current state of the health system based on a set of state variables and a dynamic and adaptive statistical analysis of patient flow and hospital LoS.
- The combination of all elements in a DES model flexible enough to recreate scenarios based on stochastic models fitted to data (data-driven prediction), scenarios defined by expert judgment, and a mixture of both.
- In practical terms, this paper also shows how operations research can contribute to a rapid respond to a healthcare crisis by reporting on a successful real-world application of the simulation model to support a decision-making process of crucial importance to the health of patients in two Autonomous Regions of Spain (Navarre and La Rioja).

The rest of the paper is organized as follows. Section 2 offers a review of related literature dealing with the use of quantitative methods for the prediction and efficient management of health care system requirements. Section 3 studies the adequacy of PG models to predict the spread trend of a pandemic. The modeling of patient flow through the hospital is presented in Sect. 4. The structure of the DES model and the methodology used to set up the simulation are included in Sect. 5. Results of the application in the Autonomous Regions of Navarre and La Rioja (Spain) are included in Sect. 6. Finally, Sect. 7 ends the paper with the conclusions of this work.

## Related literature

Simulation is one of the most suitable analytical tools for the analysis of complex systems, such as healthcare systems, as reflected in numerous specialist articles describing the use of simulation models for decision-making in the healthcare context. DES has been used to model and analyze all aspects of logistics management in healthcare, particularly the improvement of patient flow management, bed-planning, waiting list management, health service design, medical staff scheduling, etc. For reviews of the use of simulation models in healthcare, see Brailsford et al. (2009); Günal and Pidd (2010); Katsaliaki and Mustafee (2011); and Mielczarek and Uziałko-Mydlikowska (2012). These simulation models usually focus on studying the stationary state of the health system to support strategic decisions for resource sizing or management policy design purposes.

The ultimate goal of these models is to match resource availability with demand in order to provide high-quality patient care while maintaining adequate human and technological resource provision. Some of the problems analyzed in this framework are patient flow (Shahani et al. 2008; Kolker 2009), bed planning (Ridge et al. 1998; Zhu et al. 2012; Rodrigues et al. 2018), health service design (Mallor et al. 2016), and medical staff scheduling (Erhard et al. 2018), among others. Despite reports in the medical literature of discrepancies between assumptions in mathematical simulation models and the behavior of real healthcare systems (Azcarate et al. 2020), there is no doubt about the usefulness of simulation models for the analysis of relevant problems in complex healthcare systems.

However, simulation not only helps to ensure the highest quality healthcare in terms of staff and facilities, it also improves the delivery of best practice. Since the pandemic began, all national governments and the WHO have extensively used simulation modelling to identify the best strategies for reducing the impact of COVID-19. Currie et al. (2020) identify challenges from this disease and discuss how simulation modelling can help decision-makers to make the best informed decisions.

The accuracy of a simulation model for the prediction of resource needs during a pandemic is dependent upon the design of an accurate model to forecast patient arrivals at the health facility. Most infectious disease prediction methods rely on differential equation models based on population dynamics (Grassly and Fraser 2008; Brauer and Castillo-Chavez 2012). These mathematical models are essential for understanding the course of the epidemic and planning effective control strategies (Anderson and May 1991; Diekmann and Heesterbeek 2000; Hethcote 2000). One of the most widely used models of human-to-human transmission is the SIR model (Kermack and McKendrick 1927). Members of the population are sorted into different status categories: S (Susceptible), I (Infected), and R (Remove). The portion of population in each state is calculated over time by estimating the rate of transition from one state to another. With more complex model specifications, it is possible to recreate the spread of a specific epidemic. Extensions of the classical SIR model (Anastassopoulou et al. 2020; Giordano et al. 2020; Lin et al. 2020; Zhou et al. 2020; Casella 2021), as well as stochastic transmission models (Hellewell et al. 2020; Kucharski et al. 2020) have indeed been developed for the COVID-19 pandemic. However, such models are complicated and need strong assumptions and simplifications, because they are based on a set of differential equations with initial conditions and a number of adaptive parameters (Xia et al. 2009; Li et al. 2014; Magal et al. 2016; Li and Zhang 2017). Reliable values of those parameters only become available at the end of the pandemic and they depend on non-pharmaceutical interventions dictated by political decisions. There is also a need for other mathematical models that can be adapted to daily pandemic data.

PG models provide a simpler alternative for modelling the number of cumulative positive cases, hospitalizations and other pandemic variables. Growth curves are used in a wide range of research areas, such as fishery research (Oliveira Zardin et al. 2019; Oribe-Pérez et al. 2020), biology (Sun et al. 2020), or other infectious disease outbreaks (Horimoto et al. 1997; Roberts and Saha 1999; Viboud et al. 2016; Ghazvini et al. 2019). Specifically, Logistic, Gompertz, Rosenzweig, and Richards models have been used to model the spread of outbreaks such as A/H1N1 and Ebola in (Liu et al. 2015). The COVID-19 research has produced several papers describing the development of a growth model to predict new cases in countries such as China (Shen 2020), India (Malavika et al. 2021), Spain (Sánchez-Villegas and Daponte Codina 2020), and other European countries (Cássaro and Pires 2020). These mathematical models present a set of mathematical equations including adaptive parameters that can be determined numerically based on available real data (Panovska-Griffiths 2020). The model can be used daily (by updating the number of positive cases) and automatically adapted to individual parameter trends.

If all the mathematical models mentioned in the previous paragraphs could be fitted to real data, it would be possible to obtain an accurate prediction of what might happen in the future (e.g., emergency planning, resource allocation) (He et al. 2020; Poston et al. 2020; Steinberg et al. 2020). This is very important; especially for typically scarce hospital resources, such as ICU beds. Manca et al. (2020) present and discuss a few regression models built on historical ICU admissions and patient death data during the COVID-19 pandemic. They are capable of reproducing the bed occupancy curve using regression models with great potential for decision-making and emergency planning in future pandemics.

In recent decades, moreover, healthcare simulation models using advanced technology have become a new experience-based learning support (Almagooshi 2015; Persson 2017) enabling healthcare professionals to acquire new cognitive, technical, and behavioural skills. Before working in real-world patient treatment scenarios, both professionals and students can benefit from this experience-based form of learning in a risk-free decision-making environment (Palominos et al. 2019). Simulation models of the type presented in this paper also enable training in the management of health care services during emergencies. When resources are in short supply, one of the most critical decisions is how to allocate them to patients, especially when they can mean the difference between survival and death, as is the case with ICU patients. This triage becomes even more difficult during pandemics, when resources are stretched even further. Different ICU triage protocols for use in pandemics have been suggested in Cheung et al. (2012); Christian et al. (2014); and Zhang et al. (2020). Forecasting bed demands is essential to avoid ethical dilemmas (Azcarate et al. 2020; Garcia-Vicuña et al. 2020). According to Utley et al. (2011), “the impact of triage is dependent on the level of demand and on the scale of achievable differences between included and excluded groups in terms of anticipated LoS and critical care survival”. A simulation model can improve critical resource planning during a pandemic; and can be used as an off-line learning tool to test new triage protocols, which are not always as effective as might be desired, and other hard-to-anticipate factors must be considered.

## Modelling the patient arrival pattern

In this section, we discuss the adequacy of PG models for case prediction purposes. First, we perform a statistical comparison of four different models for their suitability. We then describe the use of the Gompertz PG model to simulate daily hospitalization series.

### Population growth models

The simulation model needs to generate the daily patient arrivals to the hospital(s), which is a non-stationary process highly dependent on the number of positive (active) cases in the population. A compartmentalized epidemiological model, such as the SEIR model, enables analysis of the spread of the disease throughout a population. It models transition dynamics between four different states of a population: susceptible (*S*), exposed (*E*), infective (*I*), and recovered (*R*). The model depends on epidemiological parameters such as the infection rate (the number of people that an infective person infects per day), the disease latent time (the lag between contact with an infected person and the appearance of symptoms), the recovery rate, and the death rate. The basic SEIR model has been extended to categories such as the protected (*P*) and the quarantined people (*Q*) (Godio et al. 2020) and other case detection and symptom statuses, up to a total of eight or more compartments (Giordano et al. 2020). Stochastic transmission models have also been considered (Kucharski et al. 2020). All these extensions add details to the model but also more complexity, which does not necessarily mean greater forecasting reliability, since it increases the number of model parameters to be estimated (Roda et al. 2020). Roda et al. (2020) demonstrate a linkage between the transmission rate and the case-infection ratio, resulting in a continuum of best-fit parameter values. These can produce significantly different predictions for the epidemic: the hallmark of a non-identifiability problem. These difficulties motivated us to consider parametrically parsimonious models, such as the PG type, which are able to generate curves of the shapes generally associated with pandemic variables (positive (active) cases, hospitalizations, deaths): monotonic, humped, and S-shaped. Rypdal and Rypdal (2020) found that PG models are obtained from the SIR model by making reasonable assumptions about the SIR parameter trends over time.

Examples of growth models found in the literature include the Gompertz (Gompertz 1825), the Richards (Richards 1959), the Stannard (Stannard et al. 1985), and the Logistic model (Ricker 1979). They all start with exponential growth but each has a specific, gradually decreasing growth rate. All produce S-shaped curves describing the evolution of pandemic variables departing from one or a few initial cases, growing initially at an exponential rate before reaching a plateau, and then decreasing to zero when the pandemic expires. The equations describing the number of cases in population *y*, at time *x*, take the following form:

1 $$ {\text{Gompertz}}:\quad y\left( x \right){ } = { }a\cdot{\text{exp}}\left[ { - {\text{exp}}\left( {b - cx} \right)} \right] $$

2 $$ {\text{Logistic}}:\quad y\left( x \right) = { }\frac{a}{{\left[ {1 + {\text{exp}}\left( {b - cx} \right)} \right]}} $$

3 $$ {\text{Richard}}:\quad y\left( x \right){ } = { }a\left\{ {1 + v\cdot{\text{exp}}\left[ {k\left( {\tau - x} \right)} \right]} \right\}^{{\left( { - 1/v} \right)}} $$

4 $$ {\text{Stannard}}:y\left( x \right){ } = a\left\{ {1 + {\text{exp}}\left[ { - \frac{{\left( {l + kx} \right)}}{p}} \right]} \right\}^{{\left( { - p} \right)}} $$

We carried out two statistical analyses to elucidate the adequacy of PG models for representing and predicting the evolution of the pandemic caused by the SARS-CoV-2 virus. The first analysis evaluates the capacity of the four PG models to fit complete sets of real positive case data registered in the 20 most-affected countries during the first wave of the pandemic (as recorded in Worldometer on June 15, 2020). The results included in Appendix 1 show that the Gompertz, Richards, and Stannard models have similar goodness of fit; with all three outperforming the Logistic model. These results are consistent with Rypdal and Rypdal (2020) who found that the COVID-19 related death rate curves of most countries are well described by the Gompertz growth model. The cumulative positive case, hospitalization and death curves have similar shapes because they are all scaled by the factor $s$, and translated by the factor $t$.

5 $$ y_{h} \left( x \right) = s_{h} y_{I} \left( {x - t_{h} } \right) $$

6 $$ y_{d} \left( x \right) = s_{d} y_{I} \left( {x - t_{d} } \right) $$

where $y_{I}$, $y_{h}$, $y_{d}$ are the cumulative series of positive cases, hospitalizations and deaths, respectively; $s_{h}$, $s_{d}$ are the scaling factors for hospitalizations and deaths, respectively, and ${t}_{h}$, ${t}_{d}$ are the time lags between infection and hospitalization, and infection and death, respectively.

The second statistical analysis is designed to test the short-medium term predictive capacity of the PG models. For each of the 20 countries used in the first statistical analysis, the data up to the day on which cases exceed 25%, 40%, and 65% of the total cases registered at the end of the pandemic wave are used to predict the cases for the following successive 5, 10 and 15 day horizons. Thus, nine prediction exercises are performed for each country and each PG model. The results included in Appendix 2 show that the Gompertz model surpasses the predictive capacity of the other PG models, outperforming them in all nine cases, and being equaled by the Richards and Stannard models in only four.

Our statistical analysis supports the use of the Gompertz for modelling series of cumulative hospitalizations. The parameters of the original equation of this model, presented above, are more suited to mathematical than biological interpretation, like most equations describing sigmoidal growth curves. Therefore, before using it in our modeling, some transformation will aid interpretation of the curve. Zwietering et al. (1990) rewrite the Gompertz growth model as shown in Eq. (1).

7 $$ G\left( t \right) = A{\text{exp}}\left( { - {\text{exp}}\left( {\frac{{{\text{K}}e}}{A}\left( {D - t} \right) + 1} \right)} \right) $$

where, $e = \exp \left( 1 \right)$, $G\left( t \right)$ is the cumulative number of hospitalizations up to time $t$. $A$ is a growth model parameter corresponding to the total number of hospitalizations at the end of the outbreak. It is the upper asymptote of the curve. $K$ is the absolute growth rate of the curve at its inflection point. $D$, known as the lag time, is the time at which $G\left( t \right) = Aexp\left( { - e} \right)$, which means that it always occurs at the same percentage (6.6%) of the upper asymptote. This value is less intuitive than either of the others.

Suppose we are at pandemic day $n + 1$ and have recorded and denoted by $h\left( t \right), t = - n, \ldots , - 1$ the number of hospitalizations since the beginning and by $H\left( t \right)$ the cumulative number of hospitalizations $H\left( t \right) = \mathop \sum \nolimits_{i = - n}^{t} h\left( i \right), t = - n, \ldots , - 1$. Using these data, the Gompertz growth model parameters ${\varvec{p}} = \left( {A, K, D} \right)$ are estimated by minimizing the sum of the squared errors (SSE). The estimated parameter vector is denoted by $\hat{\user2{p}} = \left( {\hat{A},\hat{K},\hat{D}} \right)$ and the Gompertz model by $G_{{\hat{p}}} \left( t \right)$. The values of ${G}_{\widehat{p}}\left(t\right)$ are used to predict the expected number of hospitalizations for the current and following days, $t=1,\dots ,T$, as required by the simulation methodology described in the following subsection.

### Simulation of the patient arrival pattern

Once the curve $G_{{\hat{p}}} \left( t \right)$ is fitted to the hospitalization data $H\left( t \right), t = - n, \ldots , - 1$ of a certain region up to the present day, it is used to predict and simulate the number of new hospitalizations for each of the following days $t = 1 \ldots , T$. The function argument $t$ is continuous and we will assume that $t = 0$ represents both the end of the last day of recorded hospitalizations and the start of the current day. Therefore, $t = 1$ is the time at which the current day ends.

The simulation procedure is summarized in the following four steps:

1. *Fit the Gompertz curve* to cumulative hospitalization data series (e.g. by using the least squares method). Record the estimated parameter vector $\hat{\user2{p}} = \left( {\hat{A},\hat{K},\hat{D}} \right)$ and its covariance matrix ${\varvec{\varSigma}}_{{\hat{\user2{p}}}}$.
2. *Simulate a parameter vector* ${\varvec{p}} = \left( {A, K,D} \right)$ from the Multivariate Normal distribution $N\left( {\hat{\user2{p}},{\varvec{\varSigma}}_{{\hat{\user2{p}}}} } \right)$.
3. *Calculate the expected number of arrivals* $N\left( t \right)$ for day $t, t = 1, \ldots ,T$, where $t = 1$ represents the current day (start of the simulation) and $T$ is the simulation horizon. Use the Gompertz curve $G_{p} \left( t \right)$ with parameters ${\varvec{p}}$ to calculate $E\left( {N\left( t \right)} \right) = G_{{\text{p}}} \left( t \right) - G_{{\text{p}}} \left( {t - 1} \right)$.
4. *Simulate the number of hospitalizations*, for each day $t, t = 1, \ldots ,T$, in the future, as observations from a Poisson distribution with mean $\lambda \left( t \right) = E\left( {N\left( t \right)} \right)$:
  8 $$ P\left( {N\left( t \right) = k} \right) = \frac{{e^{{ - \left( {\lambda \left( t \right)} \right)}} \left( {\lambda \left( t \right)} \right)^{k} }}{k!} t = 1, \ldots ,T $$
5. *Repeat steps 2–4* as many times as necessary for the different hospitalization sequences to be simulated.

**Fig. 1:**
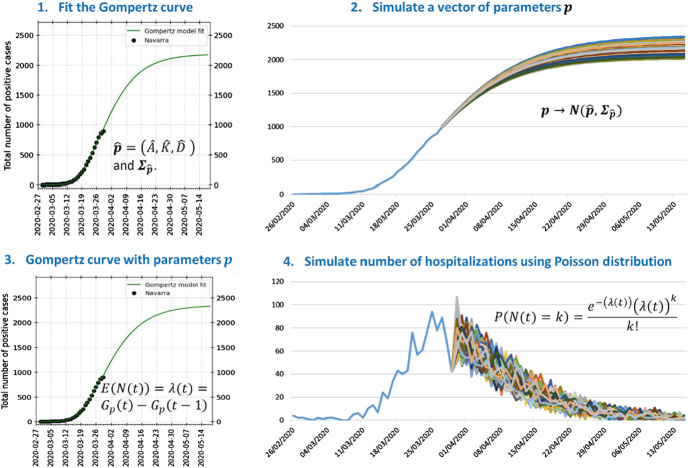
The simulation procedure for the patient arrival pattern. Steps 2–4 are replicated as often as necessary for the different patient arrival and hospitalization sequences to be simulated

This simulation procedure takes into account both variability due to uncertainty in the estimation of the Gompertz parameters and variability in hospital arrival numbers around the mean. Figure 1 illustrates the four steps. The upper-left hand corner of the graph shows the Gompertz model (green curve) fitted to the available data (black dots); the point estimator $\hat{\user2{p}}$ and the covariance matrix ${\varvec{\varSigma}}_{{\hat{\user2{p}}}}$ are used in the second step to sample a parameter vector ${\varvec{p}}$. The upper-right hand corner of the graph shows the Gompertz curves associated with parameter vectors ${\varvec{p}}$ in a tolerance region obtained from the multivariate normal distribution (Dong and Mathew 2015) as

9 $$ R = \left\{ {{\varvec{p}}{|}\user2{ }\left( {{\varvec{p}} - { }\hat{\user2{p}}} \right)^{\prime } {\varvec{\varSigma}}_{{\hat{\user2{p}}}}^{ - 1} \left( {{\varvec{p}} - { }\hat{\user2{p}}} \right) \le \chi_{3}^{2} \left( q \right)} \right\} $$

where $\chi_{3}^{2} \left( q \right)$ denotes the $qth$ percentile of a chi-square distribution with df = 3. Clearly, $R$ is the central $100q\%$ region of the multivariate normal distribution $N\left( {\hat{\user2{p}},{\varvec{\varSigma}}_{{\hat{\user2{p}}}} } \right)$.

Each parameter vector ${\varvec{p}}$ in the region $R$ is associated with a Gompertz curve $G_{p} \left( t \right)$ (shown in the lower left hand corner of Fig. 1) compatible with the observed data (and different from $G_{{\hat{p}}} \left( t \right)$). This Gompertz curve $G_{p} \left( t \right)$ provides the expected number of hospitalizations among simulated arrivals generated by sampling from a Poisson distribution. A sequence of trajectories with the simulated number of hospitalizations for future days $t = 1, \ldots ,T$ (shown in the lower right hand corner of Fig. 1) is obtained by replicatimg the sampling of a vector ${\varvec{p}}$, the calculation of the expected number of future arrivals $\lambda \left( t \right), t = 1, \ldots ,T$, from the Gompertz curve $G_{p} \left( t \right)$, and the simulation of simulated arrivals $N\left( t \right), t = 1, \ldots ,T$, from the Poisson distribution. The Gompertz curve is refitted after every new observation and the simulation of future arrivals is carried out again following steps 1 to 5.

## Modelling the patient flow

This section focuses on modelling patient flow through the health system. First, we describe the possible patient pathways through the hospital, and then explain how the LoS of each patient is modelled.

### Hospital patient pathway

COVID-19 patients can access the health system in a variety of ways: following diagnosis with COVID-19 in a primary healthcare facility, hospital emergency department, or nursing home; or after undergoing a SARS-CoV-2 test control (such as a *Polymerase chain reaction* (*PCR*) test), etc. Depending on the severity of his/her condition, the person is admitted to the health care system as a COVID-19 patient, either in a hospital ward or directly in the ICU.

**Fig. 2:**
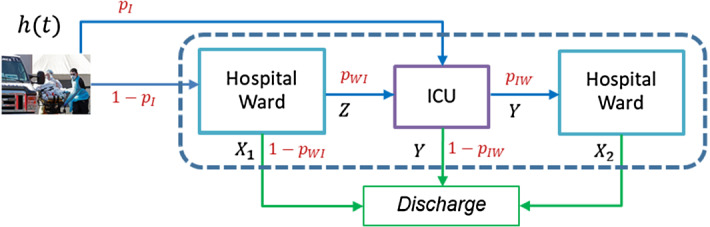
Representation of COVID-19 patient flow in the health system

The COVID-19 patient pathway through the hospital is the same as for other hospital patients, but, due to the highly contagious nature of the virus, COVID-19 patients require dedicated resources and cannot be mixed with other patients. Figure 2 shows the patient flow through the health system, highlighting the transitions between the hospital ward and the ICU. Patients can be admitted either to the ICU or first to a hospital ward with potential transfer to the ICU if their condition deteriorates. Discharge from a ward can follow either death or a health improvement. Patient transfers from the ICU to a hospital ward occur after a health improvement.

### Stochastic modelling of hospital LoS

The following variables are used to model LoS in the hospital:

- $X_{1}$, the LoS in the hospital ward of a patient not needing ICU.
- $Y$, the LoS of a patient in the ICU.
- $Z$, time spent by a patient in the hospital ward before transfer to the ICU (applies only to patients transferred to the ICU from the hospital ward).
- $X_{2}$, the LoS of a patient in the hospital ward after being discharged from the ICU.

The following probabilities determine the patient-pathway through the healthcare facilities:

- $p_{I}$, the probability of direct admission to ICU upon arrival.
- $p_{WI}$, the probability of a patient initially admitted to a ward requiring transfer to ICU.
- $p_{IW}$, the probability of patient transfer from ICU to a ward. Then, $1 - p_{IW}$ is the probability of death in the ICU.

Then, the probability of ICU requirement is $p_{I} + (1 - p_{I} )p_{WI}$.

As the pandemic progresses and more COVID-19 patients need hospitalization, the new data collected from these patients can be used to update the probability distribution parameters estimates and patient pathway probabilities. Given that only a small percentage of ward admissions and an even smaller percentage of ICU admissions have been discharged after a few weeks from the start of the outbreak, the associated information on most of them is partly unknown, that is, they constitue censored data. For example, a patient admitted to the ICU 10 days ago provides an ICU LoS datum $x$ such that $x > 10$.

This motivates us to perform daily updates of the distribution parameters and probabilities by adding the fresh data, thereby enlarging the sample size, reducing the degree of censorship bias and ultimately obtaining more accurate parameter estimates. The parameter estimation is done by the maximum likelihood method. For example, for the estimation of $\theta_{Y}$, the parameter vector of the distribution function of variable $Y$ is performed by maximizing the following likelihood function:

10 $$ L_{Y} \left( {\theta_{Y} \left| {y\left( t \right)} \right.} \right) = \mathop \prod \limits_{i = 1}^{{n_{Y} }} f_{{\theta_{Y} }} \left( {y_{i} } \right)\mathop \prod \limits_{i = 1}^{{n_{{Y^{*} }} }} \left( {1 - F_{{\theta_{Y} }} \left( {y_{i}^{*} } \right)} \right) \to \theta_{Y} = \arg \mathop {\max }\limits_{{\theta_{Y} }} L_{Y} \left( {\theta_{Y} \left| {y\left( t \right)} \right.} \right) $$

where $\left\{ {y_{i} , i = 1, \ldots ,n_{Y} } \right\}$ is the set of exact value observations, that is, those corresponding to the LoS of patients now discharged from the ICU, and $f_{{\theta_{Y} }} \left( {y_{i} } \right)$ is the density function. $\left\{ {y_{i}^{*} , i = 1, \ldots ,n_{{Y^{*} }} } \right\}$ is the set of censored values, that is, those corresponding to the LoS of patients remaining in the ICU at the time of the statistical analysis, and $F_{{\theta_{Y} }} \left( {y_{i} } \right)$ is the cumulative distribution function.

The use of probability plots facilitates identification of the parametric probability distribution family that best fits the data. The parameters of the selected probability distribution family are estimated by the maximum likelihood method. Weibull and Lognormal distribution families have proved to be good probability models for LoS variables, as will be shown in Sect. 6.

At the beginning of a new pandemic, there is insufficient understanding of the disease and possibly no known effective treatment, as was the case with the COVID-19 outbreak. As medical and biological research progresses, the discovery of new drugs and therapeutic protocols improves patient care and alters lengths of stay in hospital wards or ICUs. This observation reinforces the need to gather every possible new piece of patient admission and discharge data for use in updating estimated distribution parameters and branching probabilities.

## The discrete event simulation model

In this section, we present the mathematical modelling of hospital dynamics using a DES model. We pay attention to starting the simulation from the current state of the health system, which is one of the distinguishing features of this application of healthcare system simulation modelling.

### Entities, state variables, and events

DES models create moving entities that are transformed by several processes until they exit the modelling system. In the healthcare system that concerns us, the entities are the COVID-19 patients and the processes are the health care received in the hospital ward and/or ICU. The system is described by a set of state variables, which provide at any time a complete representation of the simulated system, and the set of events, which modify the value of the state variables. The simulation model represents patient flow through the different hospitalization routes; that is, the area enclosed by dashed lines in Fig. 2. In this subsection, we present two types of healthcare system state variables and the set of events separated into two categories.

We consider two distinct types of state variables: global and patient-level. The two global variables, $B\left( t \right) = \left( {B_{W} \left( t \right), B_{I} \left( t \right)} \right)$, describe bed occupancy by COVID-19 patients in hospital wards and the ICU, respectively, at any time $t$. Total COVID-19 hospitalizations at time $t$, $N\left( t \right)$, is given by the sum of these two state variables, $N\left( t \right) = B_{W} \left( t \right) + B_{I} \left( t \right)$.

Each patient $i$ admitted to hospital has two associated state variables. The patient-level state variable $S_{i} \left( t \right)$, which records the condition of patient $i$ at time $t$, can take one of three values: $W_{1}$, when patient $i$ is admitted to a ward without a previous stay in ICU; $I$, when patient $i$ is admitted to ICU; and $W_{2}$, when patient $i$ is in a ward after transferral from ICU. The patient-level state variable $R_{i} \left( t \right)$ records the time at which patient $i$ enters state $S_{i} \left( t \right)$.

Two different types of events can affect the values of the state variables. They have been classified by the nature of the variation in $B\left( t \right)$: an increase or decrease in $N\left( t \right)$, or a variation in $B_{W} \left( t \right)$ and $B_{I} \left( t \right)$ not affecting their sum. The first set of events $E_{A}$ are associated with patient arrival times. This group includes only external arrivals, i.e., positive cases detected outside the hospital that require hospitalization. These events are generated by the simulation methodology described in Subsection 0, and each arrival is classified as an ICU arrival or a ward arrival with probabilities $p_{I}$ and $1 - p_{I}$, respectively. The last type of patients are also subdivided into two groups, those who will require ICU admission after some time on the ward (with probability $p_{WI}$) and those who will not (with probability $1 - p_{WI}$).

The second category includes the events $E_{B}$ leading to a patient’s end of a stay in the ICU or the ward, and altering the value of their patient-level state variables, and also either $B_{W} \left( t \right)$, or $B_{I} \left( t \right)$ or both. As stated in Sect. 4, there are several events of this type:

- $E_{BZ}$ events dictating end of ward stay prior transfer to ICU, which are generated by sampling from the variable $Z$. The variable $B_{W} \left( t \right)$ decreases by one unit and $B_{I} \left( t \right)$ increases by one.
- $E_{{BX_{1} }}$ events signaling end of ward stays with no need for ICU transfer, which are generated by sampling from the variable $X_{1}$. The variable $B_{W} \left( t \right)$ decreases by one unit.
- $E_{{BX_{2} }}$ events associated with end of ward stay for a patient transferred from ICU, and generated by sampling from the variable $X_{2}$. The variable $B_{W} \left( t \right)$ decreases by one unit.
- $E_{BY}$ events associated with end of ICU stay and generated by sampling from the variable $Y$. The variable $B_{I} \left( t \right)$ decreases by one unit. The patient is transferred to a ward with probability $p_{IW}$, and $B_{W} \left( t \right)$ increases by one unit.

The event calendar vector at time $t$ has $B_{W} \left( t \right) + B_{I} \left( t \right) + 1$ positions. One includes the time of the next patient arrival (associated with event $E_{A}$), $B_{W} \left( t \right)$ positions, one for each ward patient, containing their hospital discharge times (associated with events $E_{{BX_{1} }}$ or $E_{{BX_{2} }}$) or ICU transfer times (associated with event $E_{BZ}$), and $B_{I} \left( t \right)$ positions, one for each ICU patient, storing the discharge time from ICU.

**Fig. 3:**
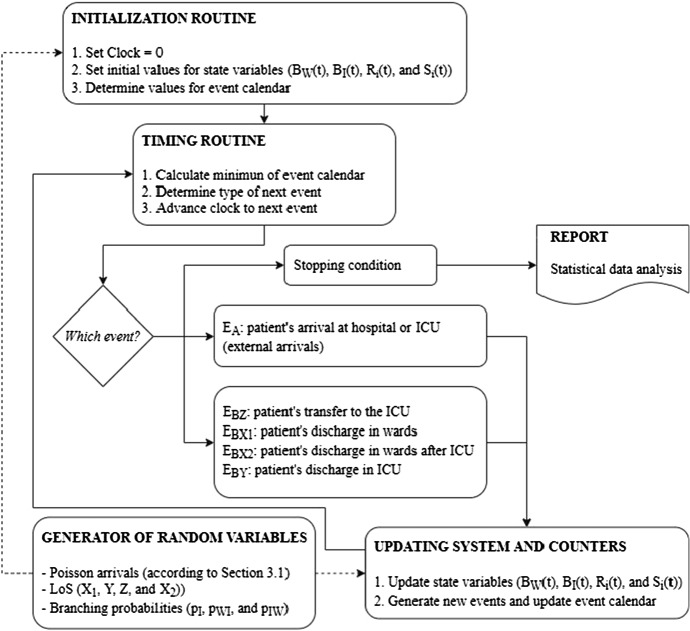
Flow diagram of the health system simulation model. Two types of events are considered, external arrivals and ward or ICU end of stays

Figure 3 outlines the DES model of the health system.

### Starting the simulation run

The purpose of the simulation model is to predict short-term resource needs, with precision strongly depending on the model’s accuracy both in representing the initial state of the healthcare system and the initial resource utilization rate. This last aspect of mathematical modeling is usually not very important when the aim of the simulation is to investigate the long-term behavior of a system in its stationary state, which is usually independent of its initial state. However, when the simulation is used as a predictive tactical decision-making support tool, the initial state of the simulation model and the initial dynamics of the system are the main determining factors of the state of the healthcare system in the near future.

The simulation clock is set to zero at the time of the last update of the Electronic Health Record (EHR)-file, which we assume to have taken place at the end of the $$kth$$ day of the pandemic. The simulation model begins to simulate future changes from day $$\left( {k + 1} \right)th$$, using the information collected during the first $$k$$ days of the pandemic, taking hospitalizations at the end of the $$kth$$ day as the initial state. The point of transition from the past to the future occurs at the beginning of the simulation run, for which the event calendar must be initialized (Law 2014). The simulation of event type $$E_{A}$$, a new patient arrival, was explained in Subsection 0. We will now explain how to simulate type $$ E_{B}$$ events for patients currently admitted, that is, at time zero in the simulation model. The value of the state variables, number of ward patients, $$B_{W} \left( 0 \right)$$, and number of ICU patients, $$B_{I} \left( 0 \right)$$, as well as times $$R_{i} \left( 0 \right)$$ in the current state $$S_{i} \left( 0 \right)$$ for each patient $$i$$, can be calculated from the EHR-file, which records admission, discharge, and ward/ICU transfer dates for each patient. This set of state variables defines the initial state of the healthcare system simulation model.

The discharge time of each ICU patient $$i$$ is calculated by sampling from the random variable $$Y$$ conditioned to a stay longer than $$r_{i} = (k + 1 - R_{i} \left( 0 \right))$$, the number of days already spent in the ICU. Let $$y_{i}$$ be a value sampled from the conditional distribution $$Y|Y > r_{i}$$, then the value $$y_{i} - r_{i}$$ is the simulated ICU discharge date for patient $$i$$, which is assigned to the position $$ E_{BI}$$ of the event calendar vector associated with patient $$i$$.

A patient admitted to a ward for $r_{i}$ days can ultimately be discharged from the hospital or transferred to ICU. The probability of ICU transfer for a patient hospitalized for $r_{i}$ days, denoted by $p_{{ICU|r_{i} }}$, is calculated with Bayes theorem:

11 $$ \begin{aligned} p_{{ICU|r_{i} }} & = P\left( {B|r_{i} days in ward} \right) = \frac{{P\left( {Z > r_{i} |B} \right) P\left( B \right)}}{{P\left( {r_{i} days in ward} \right)}} \\ & = \frac{{P\left( {Z > r_{i} |B} \right) P\left( B \right)}}{{P\left( {Z > r_{i} |B} \right) P\left( B \right) + P\left( {X_{1} > r_{i} |C} \right) P\left( C \right)}} \\ & = \frac{{\left( {1 - F_{Z} \left( {r_{i} } \right)} \right)p_{WI} }}{{\left( {1 - F_{Z} \left( {r_{i} } \right)} \right)p_{WI} + \left( {1 - F_{{X_{1} }} \left( {r_{i} } \right)} \right)\left( {1 - p_{WI} } \right)}} \\ \end{aligned} $$

where $B$ is the event of a ward patient requiring ICU transfer. $C$ is the event of a ward patient not requiring ICU transfer.

A hospital trajectory is simulated for each patient $i$ already admitted to a hospital ward. The first step of the simulation concerns the decision as to whether the patient will be admitted to the ICU (with probability $p_{{ICU/r_{i} }}$) or not (with probability $1 - p_{{ICU/r_{i} }}$). Time to ICU transfer is then simulated by sampling from the conditional distribution $Z|Z > r_{i}$, and assigning the value $z_{i} - r_{i}$ to the event $E_{BZ}$. If the patient $i$ does not require ICU care, the hospital discharge event $E_{{BX_{1} }}$ will occur at time $x_{i} - r_{i}$, where $x_{i}$ is sampled from the conditional distribution $X_{1} |X_{1} > r_{i}$.

Similarly, for each ward patient $i$ previously discharged from the ICU, the time of discharge from the hospital is simulated by sampling a value $x_{i}$ from the conditional variable $X_{2} |X_{2} > r_{i}$. The value $x_{i} - r_{i}$ is the simulated discharge time and is assigned to position $E_{{BX_{2} }}$ of the event calendar vector associated with patient $i$.

Once discharge times and transfer times between ward and ICU have been simulated for each hospitalized patient, and recorded in the event calendar (together with the arrival time of the next COVID-19 patient) the DES model is ready to advance the simulation clock from time zero to the minimum of the times recorded in the event calendar. The state variables and calendar events are then updated accordingly and the main loop of Fig. 3 is repeated until the simulation run is complete.

The fitted Gompertz curve forecasts daily patient arrivals, which can be uniformly distributed over the following 24 h or according to a non-stationary pattern when, for example, arrivals drop significantly overnight.

### Simulation output

The DES model works by generating patient arrivals, discharges, and transfers causing variations in ward and ICU occupancy levels, which are recorded by statistical counters. The simulation model includes two sources of randomness: the number of patient arrivals (hospital and ICU), and patients’ LoS. Therefore, each time the DES model is run departing from the current situation of the healthcare system (generating randomness based on a different seed) the ward and ICU bed requirements differ. Figure 3 in Sect. 5.1 shows one iteration of the simulation, with which different trajectories can be obtained with each replication of that routine. Thus, the simulation model is run many times (thousands) to get a statistical distribution of the number of ward and ICU beds needed each day.

**Fig. 4:**
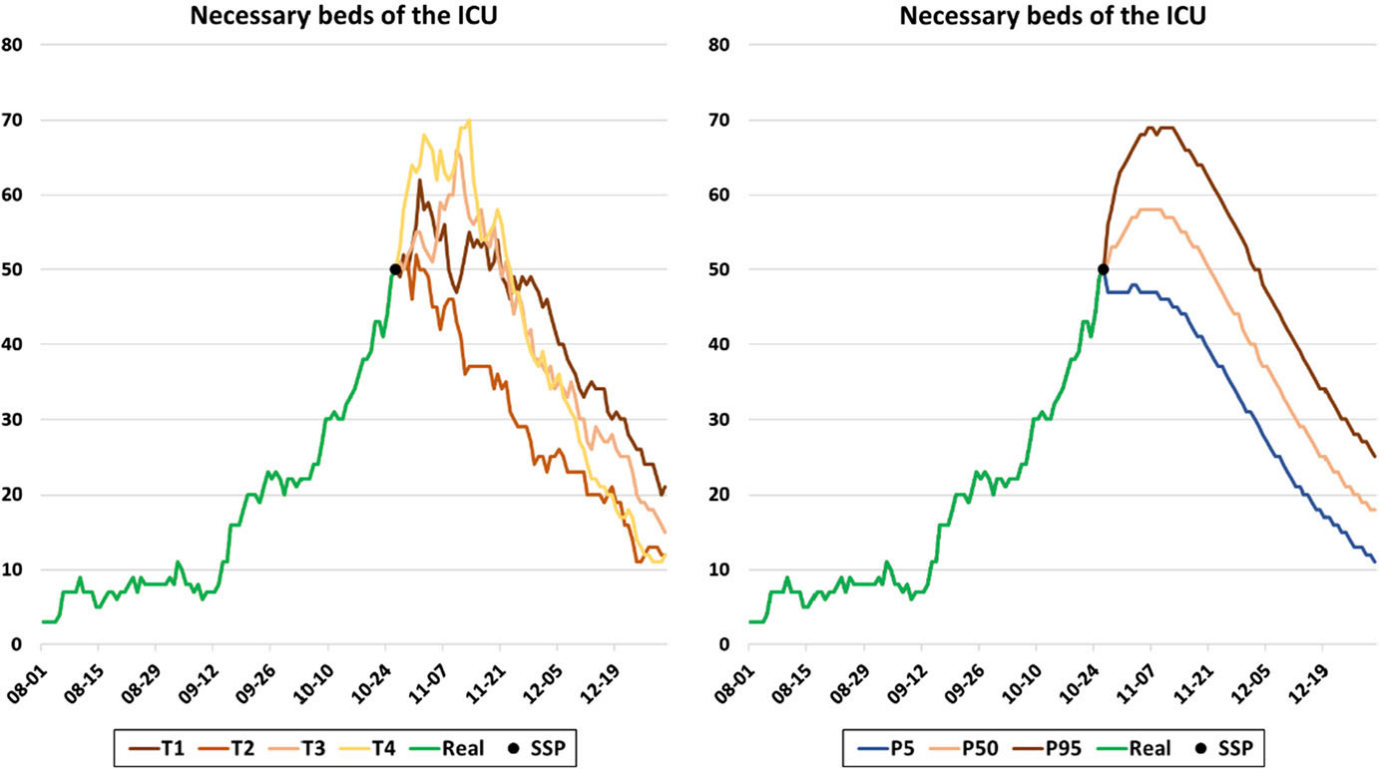
Simulation output for ICU bed demand for the following days. The left-hand side shows four different trajectories starting from the SSP; the 3 lines on the right-hand side correspond to the 5th, 50th, and 95th percentiles

The DES simulator generates percentile data, which are stored in an Excel file. The 5^th^ percentile (P5), 50th percentile (P50), and 95th percentile (P95) are plotted on a graph as confidence bands for future resource needs. Figure 4 shows an example of these graphic outputs. The green line represents the real occupancy trend and the black dot indicates the SSP, that is, the moment from which the hospital system dynamics are simulated. The left-hand side shows four different possible ICU bed demand trajectories (T1, T2, T3, and T4), while the right-hand side shows the confidence bands.

## Application of the DES model

The methodology introduced in the previous Sections, implemented in software, has been used by the Governments of the Spanish Autonomous Regions of Navarre and La Rioja to support bed planning in their hospitals during the two pandemic waves experienced to date. We briefly describe how the virus has affected these two regions globally, then explain the stochastic modelling of the hospitalized patients and their pathway through the hospital, and then present the predictions obtained by the DES model at different times. We conclude this Section with some observations and tips for the practical use of this forecasting tool.

### Incidence of COVID-19 disease

Navarre and La Rioja are two Regions of northern Spain with populations of about 650,000 and 350,000, respectively, more than half concentrated around the capitals (Pamplona and Logroño). With this population distribution, Navarre Health Services have a main hospital in Pamplona, with a bed capacity of more than 1000, and two secondary hospitals in two of the most populated cities (Estella and Tudela) bringing total bed capacity to 1,466 ward beds and 45 ICU beds. La Rioja has a main hospital in Logroño with 630 hospital beds and 21 ICU beds and a secondary 80-bed hospital in Calahorra. Both regions have the possibility of increasing bed numbers if necessary.

**Fig. 5:**
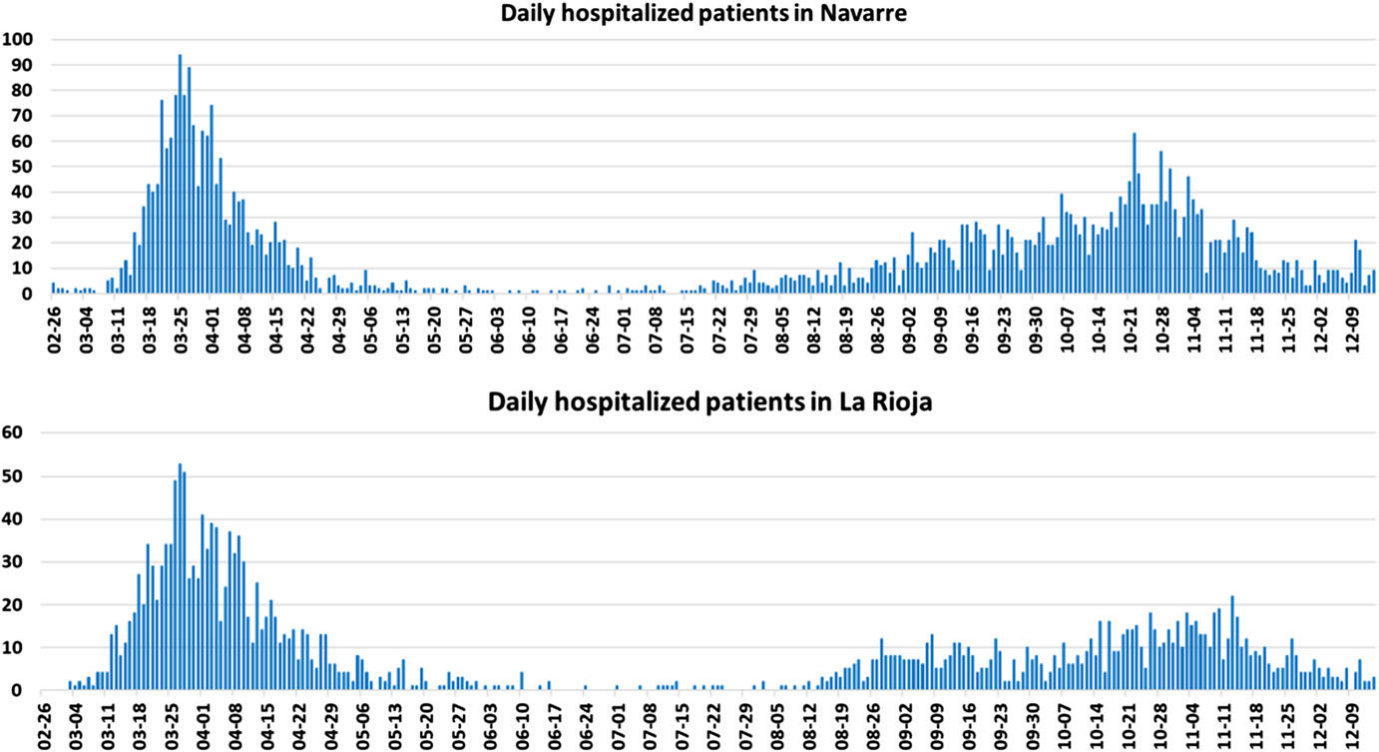
Daily recorded hospitalizations in Navarre and La Rioja from early March 2020 to mid-December 2020

Navarre and La Rioja figure among the five Spanish autonomous regions with the highest cumulative COVID-19 rates during both waves of the pandemic, according to data collected by the Governments of Navarre and La Rioja. Daily numbers of new admissions have important implications for hospital management teams. Figure 5 shows hospital admission statistics for both regions from early March 2020 to mid-December 2020. Two waves can be appreciated each with its own characteristics. The first is shorter but steeper, while the second is more prolonged. By December 16, 4228 COVID-19 patients had been admitted to hospitals in Navarre (6.5 per 1,000) and 2253 COVID-19 patients in La Rioja (6.4 per 1000).

### Stochastic modelling of hospitalizations and lengths of stay

**Fig. 6:**
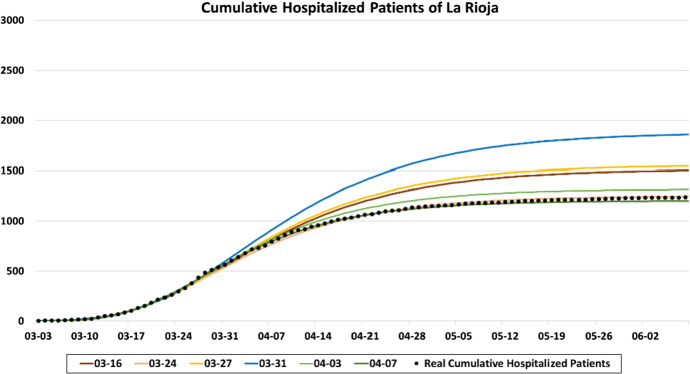
Cumulative hospitalizations in La Rioja from March 3 to June 9, 2020, and different curve fits obtained from the Gompertz growth model

**Fig. 7:**
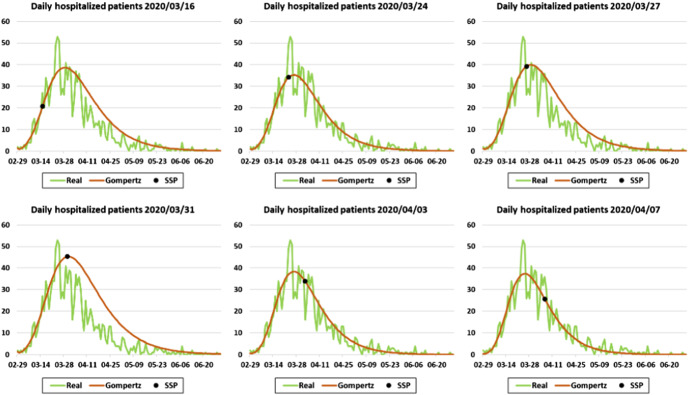
Six different curve fits obtained from the Gompertz growth model compared with the daily hospitalization series for La Rioja from March 3 to June 30, 2020

As the pandemic spreads, the data load increases, making it possible to improve the simulation model. Since March 16, 2020, the arrival pattern is calculated from the hospital admission series. Figure 6 shows different results after fitting the Gompertz growth model to cumulative hospitalizations in La Rioja during the first wave. As the pandemic progresses and more data becomes available, the Gompertz curve fit usually improves. However, due to the wide variability of the real data, minor fit deviations, such as that of March 31, 2020, are possible. Separate curve fits are shown in Fig. 7, along with the real daily hospitalization series. These graphs show the wide variability of the data.

**Fig. 8:**
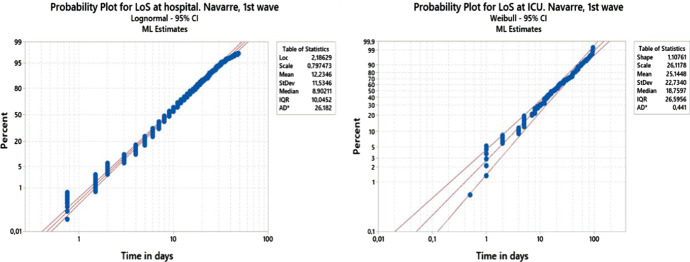
Probability plots of the fits of the ward and ICU LoS distributions during the first wave of the pandemic in Navarre

Both ward and ICU lengths of stay are estimated daily, as explained in Sect. 0. Ward lengths of stay ($X_{1}$) fit reasonably well to a lognormal distribution ($LN\left( {\mu ,\sigma } \right)$), whilst the Weibull ($W\left( {\alpha ,\beta } \right)$) distribution is found to provide a better fit for ICU LoS ($Y$). During the pandemic, each time the data was analyzed, the distribution parameters were reset for the simulation. Table 1 lists the probability distributions that best fit the lengths of stay in the two waves for Navarre (Na) and La Rioja (Ri), sorted by gender, male (M) and female (F). $N$ stands for the number of patients analyzed. Differences can be observed between regions, waves, and genders, especially in ICU lengths of stay and the percentages of ICU admissions. Figure 8 shows two probability plots obtained from the fits of the ward and ICU LoS distributions (regardless of gender) during the first wave of the pandemic in Navarre.

**Table 1:** The parameters fitted to different populations at different moments during the pandemic, sorted by region (Navarre and La Rioja), wave, and gender, showing ward and ICU lengths of stay distributions and ICU admission probabilities

Region	Wave	Gender	N	$X_{1}$(days)	$\overline{{X_{1} }}$(days)	$Y$(days)	$\overline{Y}$(days)	$p_{I}$	$p_{wI}$	$p_{IW}$
Na	1st	M	929	LN (2.220; 0.845)	13.148	W (30.191; 1.184)	28.501	0.026	0.074	0.682
Na	1st	F	807	LN (2.131; 0.819)	11.781	W (18.304; 1.055)	17.919	0.011	0.040	0.683
Na	2nd	M	1,313	LN (2.021; 0.792)	10.325	LN (2.550; 1.075)	22.815	0.021	0.095	0.678
Na	2nd	F	1,189	LN (1.970; 0.834)	10,151	LN (2.427; 0.876)	16.613	0.020	0.049	0.859
Ri	1st	M	662	LN (1.882; 0.875)	9.630	W (14.385; 1.028)	14.226	0.039	0.063	0.455
Ri	1st	F	583	LN (1.843; 0.779)	8.555	W (14.196; 1.038)	13.986	0.012	0.031	0.480
Ri	2nd	M	559	LN (1.965; 0.774)	9.625	W (25.719; 1.166)	24.381	0.081	0.115	0.769
Ri	2nd	F	450	LN (1.874; 0.805)	9.002	W (14.283; 1.184)	13.483	0.044	0.035	0.800

### Ward and ICU bed occupancy forecasts

**Fig. 9:**
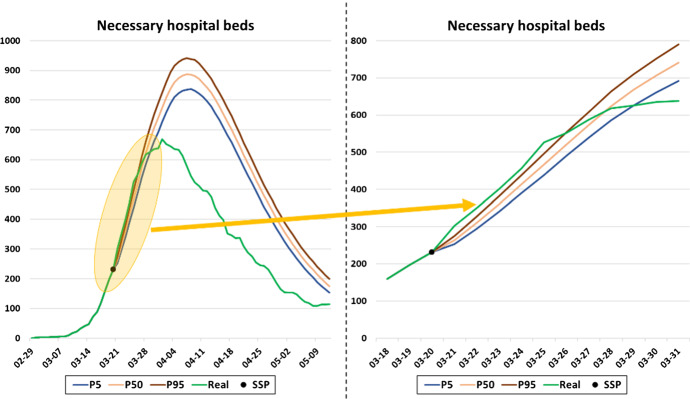
The prediction made on March 21, 2020 for bed occupancy in the hospitals of Navarre and the real occupancy levels. The area shaded yellow highlights the ability of the simulator to obtain accurate 10-day occupancy forecasts

**Fig. 10:**
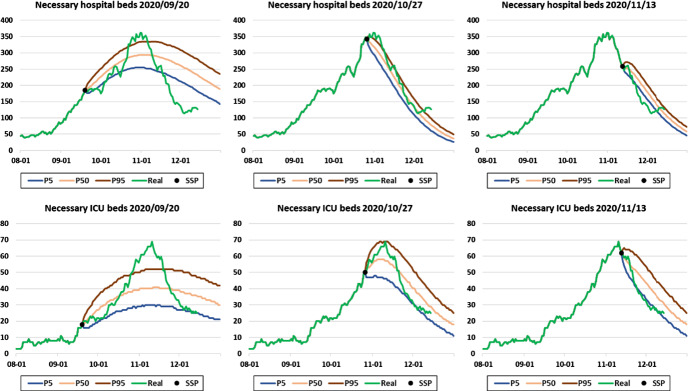
Comparison between the predictions made in Navarre at different moments of the second wave (2020/09/20, 2020/10/27, and 2020/11/13) for the number of beds occupied in both hospital and ICU, and real occupancies

Figure 9 shows the bed occupancy forecasts for the hospitals of Navarre based on the March 21, 2020 simulation for the following days. Note that the most important predictions for the medical staff are for the short-medium term (yellow-colored area in Fig. 9), and there is a close match between the simulated and the real data, plotted in green. The simulator's ability to obtain accurate 10-day forecasts, even in the early stages of the pandemic, is demonstrated here. More ward and ICU bed occupancy predictions at different moments of the second wave in Navarre, in comparison with real occupancy can be seen in Fig. 10. Three dates have been selected to show the data trend pattern. The first is September 20, 2020, when the occupation began to increase significantly. The second is October 27, 2020, some days before the peak in ward and ICU bed occupancy. The last is November 13, 2020, when peak occupancy had passed and a downward trend had begun. The results were derived from the 2000 simulation runs conducted for each date.

### Tips for the use of the DES model in practice

All results shown in the previous subsections were obtained by fitting the growth model and probability distributions to the available data at the prediction times. However, during the first stages of an outbreak, when patient hospitalization data are scant, it could be hard to achieve accurate Gompertz model parameters and LoS probability distribution estimates to feed the simulation model. The beginning of an outbreak is usually marked by exponential growth in the data, potentially leading to a very high upper asymptote from the Gompertz model, which, in practical terms, could be considered as infinity (e.g. several orders of magnitude greater than the total population of the region). Taking this estimation as a simulation input, bed demand rises exponentially to figures much higher than the entire regional population. This is not a realistic estimation even in the worst case scenario of the entire population being infected. Nevertheless, in this case, the estimation would be valid for as many days as the exponential growth holds, and, as more data is collected, the accuracy of the upper asymptote estimate increases.

However, to avoid unlimited exponential growth, and improve the accuracy of the estimates at the beginning of a new pandemic wave, we recommend conducting a mixed estimation of the Gompertz parameters, combining an estimate based on expert opinion for one parameter with statistical fit estimates for the other two. Specifically, experts are able to estimate total hospitalizations based on the population incidence rate scaled by a hospitalization factor. For example, at the beginning of the first wave of the pandemic, Navarre Health Administration professionals guessed that 1% of the population would catch the virus (based on flu incidence), and 40% of the cases would require hospitalization (estimating from initial data). Using values ranging around these estimates, we could run the simulation model to obtain possible hospitalization scenarios throughout the entire wave. These predictions overestimated total hospitalizations by the end of the first wave by only 30%. At the beginning of the second wave, the initial predicted maximum can be the value observed in the first wave or a percentage of it. However, as soon as enough data are available for an accurate parameter estimation, the simulation model should be completely data-driven.

A similar problem arises when estimating the parameters of the LoS probability distributions at the onset of a new wave. When insufficient data prevents the statistical estimation of all parameters, the simulation model has to be flexible enough to allow manual parameter input. We recommend the use of the triangular distribution to represent the LoS for different hospital status levels. The triangular distribution family is a popular choice for the estimation of task completion times because it embodies the idea of the ‘three-point estimation’ where subjective judgment is used to estimate a minimum, a ‘best guess’, and a maximum value of the variable of interest (Law 2014). Experts can rely on values reported for the countries first affected by the pandemic (for example the cases of China and Italy are described in Grasselli et al. (2020); Guan et al. (2020); Young et al. (2020); and Zhou et al. (2020)). For the second and successive waves, the probability distributions estimated at the end of previous waves can be used initially. For example, during the first days of the outbreak, Navarre Health Administration experts fixed the minimum, maximum, and most probable total LoS as 10, 18, and 13 days respectively.

## Conclusion

Healthcare systems are overburdened as high demand for healthcare services from COVID-19 patients places strains on ICU capacity and creates excessive workloads for healthcare professionals. Accurate predictions of patient care resource needs are essential to advanced resource planning which can ease pressure on the system and relieve stress among hospital staff. Accurate predictions optimize response times and thus help to save lives.

Under normal circumstances, managers cope with demand surges through resource contingency plans based on predictions made about one week in advance. In this paper, we have developed a DES model to predict hospital resource needs, particularly in terms of ward and ICU beds. The simulation model is fed with new hospitalization predictions generated by a PG model. The Gompertz growth model was selected following an analysis of the fit and forecasting properties of four PG models: Logistic, Richards, Stannard, and Gompertz. Forecasting improvements could be achieved using an ensemble of these models, but such an exercise is beyond the scope of this paper and remains for future research. Forecasting accuracy can be improved by including other factors affecting resource consumption, such as age and the Adjusted Morbidity Group (AMG), in LoS stochastic models.

The structural simplicity of the simulation model makes it appropriate for general use, i.e., it can be adapted to estimate bed needs in any geographic area. The growth model requires only three parameter estimates, which can be obtained directly from the observed data. Easy online parameter estimation is one of the advantages of this model over other complex models, such as the SIR type.

It is worth mentioning the strength of simulation models in this context of uncertainty, that is, their capability to run what-if scenarios enabling decision-makers to explore the consequences of different policy choices, such as the spatial allocation and quantity of additional healthcare resources required by COVID-19 patients in a context of uncertain demand. The simulation model is data-driven, patient arrivals and lengths of stay can be estimated from data, but it also has the flexibility of allowing the use of simulation from user-determined input to explore additional scenarios.

A distinct technical/methodological feature of the simulation model is its focus on the transition period of the health system rather than the stationary state as is usual in simulation studies or on transition periods following regeneration points. This simulated transition period is unique, given that the outbreak has no regeneration points. Therefore, accurate representation of the initial health system status is paramount. The simulation of remaining LoS per hospitalization has shown to be a key point to the smooth projection of health system dynamics and the process of linking them (and mixing them) with the new dynamics obtained from simulated new patient arrivals and lengths of stay. However, the simulation of the remaining LoS depends on the amount of information known about hospitalized patients. In this paper, we have considered patient-level information (exact admission and discharge dates). In cases where only aggregated hospital-level information is available, that is, daily numbers of admissions and discharges, an estimated admission date per patient at time zero of the simulation is required.

The simulation model can be extended to include non-COVID-19 ICU and ward bed utilization. This extension would enable the creation of hospital scenarios on which to test the effects of decisions involving other hospital areas, such as a reduction in elective surgeries to free more beds for COVID-19 patients during epidemic waves. The ultimate purpose is to create a learning tool by developing an interactive simulation model to enable the inclusion of patients from all types of pathways (ordinary and non-ordinary, such as pandemic patients), where bed management decisions are made by the program user.

The methodology presented in the previous Sections was implemented in software using the Python programming language. It takes input from a data file containing a record of six variables including sex and age of patient and four dates representing the hospital and ICU admission and discharge times for each patient admitted to the hospital so far. The dates for patients still awaiting discharge or ICU admission are left blank. These four dates enable estimation of all the LoS probability distributions and branching probabilities. The additional age and sex data enable segmentation of the patient population. The functionalities and manner of use of the software evolved through time and from one pandemic wave to another. Initially (from March 2020 to June 2020), the simulation model was computationally implemented on its own, such that a daily manual statistical analysis was required to fit probability distributions. Throughout this period, the predictions and reports were drawn up by the research group and sent to the hospital’s COVID-19 logistics manager. Only one regional government used the results of our simulation model during this period. The statistical analysis was automatized and integrated in the software during the summer of 2020. In addition, the user interface, output analysis and automatic reports generation were also implemented. Then, from October 2020 and throughout the second pandemic wave, the analyses performed by local government health administration personnel (Govts. of Navarre and La Rioja), under the supervision of the research group. From December 2020, and throughout the third and fourth waves, the health administration analysts worked almost autonomously. During the last period (January to May, 2021) the research group also assisted the Spanish Health Ministry by providing predictions for each of the 17 Autonomous Communities in Spain.

After 15 months’ cooperation with health authorities, we have reached the conclusion that the success of this operations-research support system for decision-makers in difficult pandemic times is due to the following factors:

- Multidisciplinary teamwork and a background of cooperation with health managers. The research group q-UPHS (www.unavarra.es/quphs) has been cooperating for more than 10 years in the solving of real problems surrounding health service improvements. Problem analysis is always addressed through multidisciplinary teamwork involving academics (engineers and mathematicians) and health service personnel (managers, medical staff, and computer scientists).
- A request for assistance from the health administration. At the beginning of the pandemic, health managers raised the need for a a short- and medium-term bed-demand forecasting method to improve their bed management system. Medical space and equipment (including staff) planning is based on 10-bed modules. Prior knowledge of bed needs therefore facilitates resource planning.
- Rapid response. Five days after the original request, the group presented the simulation model and the initial results (predictions) for validation by the region’s hospital and healthcare logistics managers.
- Joint development of the model. Decision-makers were involved in the development of the model and maintained continuous communication with the research team.
- Continuous improvement of the computer application. Suggestions made by health managers and a user-friendly software interface were implemented to free users gradually from the need for supervision by the research team.
- Joint monitoring of the results. Quality assessment and critical analysis of the predictions were performed jointly by the research team and health managers.

Thus, the simulation paradigm presented in this paper is suitable for the realistic representation of health service processes, which makes it more credible and easier to understand by the managers who will have to rely on the results in their decision-making. From March 2020 until the moment of writing this paper (end of June 2021), the simulation model has been used daily to predict hospital resource needs in the Spanish regions of Navarre and La Rioja, and all other Spanish Communities from January, 2021. We found that the involvement and continuous improvement suggestions of the hospital logistics manager in the development of the simulation model has been crucial for obtaining a user-centered simulator and a practical forecasting tool to enable daily updates of data from the hospital administration’s information system.

## Data Availability

Historical data is available on publicly accessible web pages. The patient-level data used in the case study cannot be publicly divulged.

## Statistical analysis to elucidate the suitability of PG models to represent the evolution of the COVID-19 pandemic

The parameter estimation of the PG models is done by minimizing the sum of squared errors. There are functions implemented in free software that perform this estimation of parameters, for instance, the *curve_fit()* function in the *optimize* module of SciPy in Python or the *growthrates* package in R. The fit quality is measured by the Mean Absolute Errors (MAE). Table

**Table 2:** The 20 most-affected countries by COVID-19 until June 15, 2020. The last four columns show the MAE calculated for the fit with each of the applied models

#	Country	Population	Total positive cases (2020–06-15)	Logistic	Gompertz	Richards	Stannard
1	USA	330,922,877	2,094,069	47,128.9	**21,461.2**	21,464.4	21,466.7
2	Brazil	212,496,348	867,624	3182.3	3245.1	**2754.7**	**2754.7**
3	Russia	145,932,063	528,964	5667.4	**1688.7**	**1689.0**	**1689.2**
4	India	1,379,418,901	332,424	1392.9	**537.9**	**538.0**	**538.2**
5	UK	67,871,466	295,889	4758.0	**1046.6**	**1046.9**	**1047.2**
6	Spain	46,754,084	245,194	5676.2	**2261.7**	**2262.1**	**2262.6**
7	Italy	60,465,149	236,989	4928.0	**1061.2**	**1061.6**	**1061.7**
8	Peru	32,951,046	229,736	2399.4	**1255.3**	1256.6	1257.6
9	Iran	83,944,885	187,427	8105.8	**6176.3**	**6176.6**	**6176.9**
10	Germany	83,773,297	186,461	4091.4	**1519.2**	**1519.5**	**1519.7**
11	Turkey	84,299,464	178,239	5335.7	**2418.3**	**2418.8**	**2419.1**
12	Chile	19,109,226	174,293	1132.5	1601.2	**1068.5**	**1068.5**
13	France	65,267,844	157,220	3396.4	**1547.0**	**1547.2**	**1547.3**
14	Mexico	128,873,820	153,507	**1418.9**	1641.0	1535.5	1535.5
15	Pakistan	220,685,460	144,478	1631.5	1324.5	**1301.0**	1321.6
16	Saudi Arabia	34,788,836	127,541	1709.0	**814.9**	**814.9**	**814.9**
17	Canada	37,728,057	98,776	1494.6	**331.0**	**331.1**	**331.1**
18	Bangladesh	164,618,467	87,520	655.6	**315.5**	**315.6**	**315.7**
19	China	1,439,323,776	84,335	1166.6	1133.0	**1097.6**	**1097.7**
20	Qatar	2,807,805	79,602	417.7	369.2	**263.6**	**263.6**

Bold values represent the best scores

2 includes all MAE values calculated for each country and model. The best fits are marked in bold (differences less than 0.1% are not distinguished). Additional information in this table is the total population of each country and the total number of positive cases on June 15, 2020.

## Positive cases predictions for the following 5, 10, and 15 days, at 25%, 45%, and 65% of total cases detected

The prediction of the fitted curves for the next 5, 10, and 15 days is assessed by calculating the MAE. These time horizons are considered as sufficient for the hospital managers to adapt extra resources for new needs. As new positive case data is added every day, and predictions are refreshed also every day, the long-term predictive capacity of the model will not be analyzed. Table

**Table 3:** The number of countries in which each model is equal or better than the others in terms of predicting new positive cases for the next 5, 10, and 15 days

Model	25%			40%			65%		
	5 days	10 days	15 days	5 days	10 days	15 days	5 days	10 days	15 days
Logistic	4	5	5	4	4	3	2	1	2*
Gompertz	**11**	**12**	**13**	**13**	**14**	**14**	**15**	**15**	**13***
Richards	**11**	9	7	**13**	9	10	**15**	**15**	11*
Stannard	**11**	8	7	**13**	9	10	**15**	**15**	11*

Bold values represent the best scores

^*^These values are of 18 countries because 2 of them (Pakistan and Bangladesh) have no data for that period

3 summarizes the relevant information from all the tables shown at the end of this appendix. It indicates the number of countries in which each model is the best in terms of predictive capacity (as before, differences smaller than 0.1% have been considered equal). It is observed that the Gompertz model is the one that more accurately predicts future values, specifically in all time horizons analyzed. For this reason, the Gompertz model is recommended for the prediction of new cases of COVID-19 (Tables

**Table 4:** MAE calculated for the fit of each model at 25% of total cases detected

#	Country	Date	Total positive cases	Logistic	Gompertz	Richards	Stannard
1	USA	2020-04-12	529,951	2,233.0	554.9	**553.7**	**553.7**
2	Brazil	2020-05-16	218,223	1497.5	1099.9	**1098.4**	1100.4
3	Russia	2020-05-04	134,687	564.2	381.8	**357.2**	**357.2**
4	India	2020-05-16	85,940	601.4	**350.0**	**350.0**	**350.1**
5	UK	2020-04-12	78,991	320.6	249.7	**209.7**	**209.7**
6	Spain	2020-03-26	66,460	297.1	**147.8**	**147.8**	**147.9**
7	Italy	2020-03-24	63,927	269.7	**150.8**	156.7	156.7
8	Peru	2020-05-08	58,526	608.4	**385.0**	**385.1**	**385.1**
9	Iran	2020-04-02	47,593	1192.5	**969.6**	**969.6**	**969.7**
10	Germany	2020-03-28	48,582	**269.9**	315.8	287.4	287.4
11	Turkey	2020-04-11	47,029	639.9	**272.4**	**272.5**	**272.6**
12	Chile	2020-05-18	43,781	741.7	**732.2**	735.8	735.8
13	France	2020-03-30	40,174	163.2	**92.6**	**93.0**	94.6
14	Mexico	2020-05-06	40,186	207.8	130.5	**128.3**	**128.3**
15	Pakistan	2020-05-15	37,218	304.3	**223.5**	**223.1**	223.5
16	Saudi Arabia	2020-05-07	31,938	**194.3**	281.4	**195.2**	**195.2**
17	Canada	2020-04-14	25,663	97.0	83.7	**57.3**	**57.3**
18	Bangladesh	2020-05-18	22,268	270.9	**134.5**	**134.5**	**134.5**
19	China	2020-02-05	24,320	164.3	**114.1**	**114.1**	**114.1**
20	Qatar	2020-05-09	20,201	147.6	206.0	**139.2**	**139.2**

Bold values represent the best scores

4,

**Table 5:** Normalized MAEs obtained for each prediction and model at 25% of total cases detected

#	Country	L_5	G_5	R_5	S_5	L_10	G_10	R_10	S_10	L_15	G_15	R_15	S_15
1	USA	6.840%	2.234%	**2.090%**	**2.091%**	15.771%	1.732%	**1.558%**	**1.559%**	26.066%	**2.473%**	2.646%	2.645%
2	Brazil	5.154%	**1.565%**	**1.596%**	**1.567%**	12.003%	**3.820%**	**3.899%**	**3.827%**	20.594%	**4.942%**	5.080%	**4.956%**
3	Russia	14.866%	**7.418%**	8.145%	8.145%	27.301%	**12.043%**	13.585%	13.585%	40.983%	**15.807%**	18.525%	18.525%
4	India	6.100%	**2.053%**	**2.055%**	**2.056%**	13.805%	**5.244%**	**5.247%**	**5.248%**	24.003%	**8.760%**	**8.765%**	**8.768%**
5	UK	8.099%	1.903%	**1.089%**	**1.089%**	16.215%	3.978%	**1.343%**	**1.343%**	26.289%	6.670%	**2.387%**	**2.387%**
6	Spain	**3.502%**	10.295%	10.283%	10.284%	**9.853%**	31.939%	31.899%	31.898%	**19.188%**	65.792%	65.700%	65.691%
7	Italy	**1.865%**	10.643%	7.881%	7.881%	**5.990%**	24.930%	17.171%	17.171%	**13.465%**	44.691%	28.294%	28.295%
8	Peru	**1.806%**	3.482%	3.480%	3.480%	5.851%	**5.624%**	**5.619%**	**5.618%**	12.624%	**8.101%**	**8.092%**	**8.089%**
9	Iran	2.635%	**1.778%**	**1.777%**	**1.777%**	**2.157%**	7.030%	7.027%	7.028%	**3.204%**	16.013%	16.006%	16.007%
10	Germany	10.915%	3.847%	**2.470%**	**2.470%**	30.272%	**3.495%**	12.258%	12.258%	50.039%	**4.967%**	23.066%	23.066%
11	Turkey	15.075%	**4.756%**	**4.761%**	**4.764%**	29.363%	**6.250%**	**6.261%**	**6.268%**	45.514%	**7.633%**	**7.652%**	**7.666%**
12	Chile	5.868%	8.082%	**5.183%**	**5.183%**	10.310%	13.948%	**8.985%**	9.253%	12.393%	18.030%	**9.969%**	20.650%
13	France	9.720%	4.424%	**4.396%**	**4.302%**	**15.968%**	17.090%	16.910%	16.324%	**26.496%**	37.173%	36.735%	35.309%
14	Mexico	6.396%	**0.895%**	2.187%	2.187%	13.397%	**2.498%**	5.044%	5.044%	23.518%	**5.093%**	9.494%	9.494%
15	Pakistan	**0.886%**	4.092%	4.063%	4.089%	**1.968%**	5.827%	5.779%	5.823%	**3.048%**	10.311%	10.236%	10.303%
16	Saudi Arabia	5.616%	**2.142%**	6.813%	6.814%	12.669%	**2.608%**	14.917%	14.917%	24.360%	**2.470%**	27.770%	27.770%
17	Canada	11.915%	**3.656%**	6.871%	6.871%	24.599%	**10.482%**	16.277%	16.277%	38.866%	**19.003%**	27.603%	27.603%
18	Bangladesh	13.722%	**8.747%**	**8.748%**	**8.750%**	25.468%	**15.608%**	**15.610%**	**15.613%**	40.500%	**24.279%**	**24.283%**	**24.288%**
19	China	13.144%	**5.681%**	**5.669%**	**5.663%**	44.477%	**12.518%**	**12.528%**	**12.534%**	75.439%	**11.828%**	**11.796%**	**11.775%**
20	Qatar	8.920%	**1.879%**	11.294%	11.294%	19.084%	**5.653%**	23.360%	23.360%	31.546%	**9.641%**	37.870%	37.870%

Bold values represent the best scores

5,

**Table 6:** MAE calculated for the fit of each model at 40% of total cases detected

#	Country	Date	Total positive cases	Logistic	Gompertz	Richards	Stannard
1	USA	2020-04-23	842,629	6372.8	**1421.9**	**1422.8**	1433.3
2	Brazil	2020-05-24	347,398	2085.6	1422.1	**1413.6**	1422.7
3	Russia	2020-05-12	221,344	1316.9	**521.2**	**521.3**	**521.5**
4	India	2020-05-25	138,845	882.2	**480.3**	**480.4**	**480.5**
5	UK	2020-04-20	120,067	617.4	320.3	**257.3**	**257.3**
6	Spain	2020-03-31	104,267	351.9	438.7	**343.9**	**343.9**
7	Italy	2020-03-30	97,689	374.7	343.5	**205.8**	**205.8**
8	Peru	2020-05-18	92,273	759.3	483.2	**482.1**	**482.1**
9	Iran	2020-04-16	76,389	1,071.3	1,201.7	**1,043.0**	**1,043.0**
10	Germany	2020-04-03	79,696	486.0	339.2	**338.2**	**338.2**
11	Turkey	2020-04-17	74,193	795.4	**345.4**	**345.5**	**345.7**
12	Chile	2020-05-26	73,997	**854.4**	858.2	**854.4**	**854.4**
13	France	2020-04-04	64,338	260.8	**200.4**	219.2	219.2
14	Mexico	2020-05-15	62,527	438.7	**151.8**	**151.8**	**151.9**
15	Pakistan	2020-05-27	59,151	335.9	**276.6**	285.4	285.4
16	Saudi Arabia	2020-05-17	52,016	360.6	273.4	**256.6**	**256.6**
17	Canada	2020-04-23	40,179	405.1	**175.7**	**175.7**	175.7
18	Bangladesh	2020-05-26	35,585	434.7	**270.4**	**270.4**	270.5
19	China	2020-02-08	34,625	185.9	130.2	**126.6**	**126.6**
20	Qatar	2020-05-18	32,604	274.1	**216.8**	**216.8**	**216.8**

Bold values represent the best scores

6,

**Table 7:** Normalized MAEs obtained for each prediction and model at 40% of total cases detected

#	Country	L_5	G_5	R_5	S_5	L_10	G_10	R_10	S_10	L_15	G_15	R_15	S_15
1	USA	9.702%	**3.501%**	**3.503%**	**3.525%**	15.921%	**6.527%**	**6.531%**	**6.567%**	22.590%	**10.112%**	**10.117%**	**10.167%**
2	Brazil	**1.165%**	3.163%	2.984%	3.161%	**2.858%**	3.924%	3.617%	3.919%	6.209%	6.041%	**5.547%**	6.032%
3	Russia	3.420%	**2.303%**	**2.301%**	**2.299%**	**5.698%**	6.093%	6.090%	6.085%	**9.245%**	10.728%	10.721%	10.714%
4	India	4.669%	**1.331%**	**1.334%**	**1.333%**	9.212%	**2.274%**	**2.278%**	**2.277%**	16.059%	**3.695%**	**3.701%**	**3.700%**
5	UK	6.330%	1.005%	**0.710%**	**0.711%**	11.831%	**0.913%**	2.210%	2.210%	19.098%	**1.746%**	5.344%	5.344%
6	Spain	6.632%	**5.630%**	5.905%	5.906%	13.015%	11.927%	**11.725%**	**11.725%**	20.529%	**18.121%**	18.794%	18.795%
7	Italy	3.211%	6.294%	**1.096%**	**1.096%**	8.347%	9.938%	**1.571%**	**1.571%**	14.945%	13.137%	**4.334%**	**4.334%**
8	Peru	6.317%	**0.817%**	1.035%	1.035%	11.975%	**1.669%**	2.145%	2.146%	22.254%	**6.073%**	6.872%	6.873%
9	Iran	**0.582%**	6.097%	1.628%	1.628%	**1.757%**	8.452%	3.338%	3.337%	**3.372%**	10.640%	5.413%	5.413%
10	Germany	11.428%	**2.083%**	2.224%	2.220%	19.849%	**2.095%**	2.390%	2.381%	27.383%	**1.755%**	**1.802%**	**1.801%**
11	Turkey	7.319%	**1.436%**	**1.432%**	**1.429%**	13.559%	**3.472%**	**3.463%**	**3.457%**	19.637%	**6.500%**	**6.486%**	**6.476%**
12	Chile	**1.645%**	**1.636%**	**1.645%**	**1.645%**	5.823%	**5.639%**	5.823%	5.817%	14.435%	14.059%	14.432%	**7.991%**
13	France	**2.158%**	12.580%	6.941%	6.940%	**3.202%**	22.970%	9.453%	9.451%	**7.545%**	35.881%	10.903%	10.900%
14	Mexico	7.023%	**2.499%**	**2.501%**	**2.503%**	12.126%	**3.558%**	**3.563%**	**3.566%**	19.255%	**5.141%**	**5.149%**	**5.154%**
15	Pakistan	3.195%	1.697%	**1.489%**	**1.489%**	11.675%	**4.830%**	7.837%	7.837%	23.909%	**11.558%**	17.595%	17.595%
16	Saudi Arabia	9.921%	**4.418%**	5.822%	5.822%	15.880%	**5.955%**	8.499%	8.499%	20.963%	**5.429%**	9.512%	9.512%
17	Canada	12.339%	**6.475%**	**6.477%**	**6.478%**	19.244%	**9.745%**	**9.749%**	**9.751%**	27.788%	**14.521%**	**14.526%**	**14.529%**
18	Bangladesh	4.289%	**1.024%**	**1.025%**	**1.025%**	11.261%	**3.951%**	**3.955%**	**3.954%**	20.791%	**7.544%**	**7.550%**	**7.550%**
19	China	11.511%	10.730%	**10.225%**	**10.225%**	37.831%	**12.943%**	16.756%	16.756%	52.069%	**10.778%**	15.505%	15.506%
20	Qatar	5.565%	**1.188%**	**1.204%**	**1.211%**	9.960%	**1.348%**	**1.379%**	**1.392%**	16.349%	**1.774%**	**1.827%**	**1.849%**

Bold values represent the best scores

7,

**Table 8:** MAE calculated for the fit of each model at 65% of total cases detected

#	Country	Date	Total positive cases	Logistic	Gompertz	Richards	Stannard
1	USA	2020-05-13	1,369,964	20,036.0	**7343.8**	7,345.7	7,346.8
2	Brazil	2020-06-04	584,016	2,654.8	**1857.5**	1,908.6	1,908.4
3	Russia	2020-05-25	344,481	1,469.0	1097.0	**846.3**	**846.3**
4	India	2020-06-04	216,919	1,113.0	**511.4**	**511.6**	**511.7**
5	UK	2020-05-06	194,990	2,136.8	**593.2**	**593.2**	**593.3**
6	Spain	2020-04-10	163,472	917.7	639.9	**382.2**	**382.2**
7	Italy	2020-04-13	156,363	1,420.8	585.3	**526.1**	**526.1**
8	Peru	2020-05-31	155,671	1,757.1	**792.6**	**792.9**	**793.1**
9	Iran	2020-05-19	122,492	3,124.1	**1912.6**	**1,912.7**	**1,912.8**
10	Germany	2020-04-13	123,016	907.9	419.2	**400.3**	**400.3**
11	Turkey	2020-04-30	117,589	1,222.6	528.2	**463.3**	**463.3**
12	Chile	2020-06-04	113,628	1,011.6	1,038.2	**844.2**	**844.2**
13	France	2020-04-15	103,573	495.0	525.4	**350.4**	**350.4**
14	Mexico	2020-05-27	101,238	746.9	**237.1**	**237.2**	**237.3**
15	Pakistan	2020-06-06	93,983	918.8	**565.8**	**565.8**	**566.0**
16	Saudi Arabia	2020-05-31	83,384	683.4	453.3	**430.7**	**430.7**
17	Canada	2020-05-08	64,922	836.0	**346.9**	**346.9**	**347.0**
18	Bangladesh	2020-06-05	57,563	534.4	**310.3**	**310.4**	**310.4**
19	China	2020-02-13	59,865	841.5	**556.6**	**556.6**	**556.7**
20	Qatar	2020-05-30	52,907	352.0	199.7	**199.6**	**199.6**

Bold values represent the best scores

8 and

**Table 9:** Normalized MAEs obtained for each prediction and model at 65% of total cases detected

#	Country	L_5	G_5	R_5	S_5	L_10	G_10	R_10	S_10	L_15	G_15	R_15	S_15
1	USA	7.840%	**3.355%**	**3.356%**	**3.356%**	10.822%	**4.910%**	**4.911%**	**4.911%**	13.993%	**6.696%**	**6.698%**	**6.698%**
2	Brazil*	2.720%	**1.445%**	**1.396%**	**1.397%**	3.898%	3.799%	**3.153%**	**3.156%**	4.007%	4.631%	**3.863%**	**3.867%**
3	Russia	4.285%	1.705%	**0.901%**	**0.902%**	7.670%	**1.598%**	2.524%	2.524%	11.790%	**1.254%**	4.822%	4.822%
4	India*	4.293%	**0.859%**	**0.861%**	**0.861%**	7.276%	**0.607%**	**0.609%**	**0.610%**	8.062%	**0.575%**	**0.577%**	**0.578%**
5	UK	9.641%	**4.441%**	**4.443%**	**4.444%**	12.914%	**5.616%**	**5.618%**	**5.620%**	16.050%	**6.681%**	**6.684%**	**6.687%**
6	Spain	6.365%	**0.354%**	2.477%	2.477%	9.902%	**0.485%**	4.430%	4.430%	13.199%	**1.001%**	6.451%	6.451%
7	Italy	7.789%	**2.131%**	3.037%	3.037%	11.523%	**3.678%**	4.993%	4.993%	15.241%	**5.390%**	7.116%	7.116%
8	Peru	10.445%	**6.119%**	**6.121%**	**6.122%**	12.525%	**5.208%**	**5.210%**	**5.212%**	15.847%	**4.811%**	**4.815%**	**4.818%**
9	Iran	14.856%	**10.700%**	**10.702%**	**10.702%**	18.765%	**13.960%**	**13.962%**	**13.963%**	23.212%	**17.807%**	**17.809%**	**17.810%**
10	Germany	4.053%	2.415%	**1.864%**	**1.864%**	7.360%	2.354%	**1.473%**	**1.473%**	10.611%	2.080%	**1.118%**	**1.118%**
11	Turkey	4.858%	1.079%	**0.484%**	**0.484%**	7.419%	**0.972%**	1.333%	1.333%	10.097%	**0.817%**	2.504%	2.504%
12	Chile*	**6.750%**	9.179%	7.516%	7.517%	**11.535%**	16.538%	17.398%	17.400%	**12.420%**	18.133%	19.826%	19.827%
13	France	5.234%	**1.690%**	1.813%	1.813%	7.530%	2.840%	**2.534%**	**2.534%**	9.905%	3.784%	**3.530%**	**3.530%**
14	Mexico	5.207%	**1.082%**	**1.084%**	**1.085%**	8.908%	**1.584%**	**1.587%**	**1.588%**	11.785%	**2.536%**	**2.537%**	**2.538%**
15	Pakistan*	11.997%	**9.348%**	**9.351%**	**9.351%**	18.651%	**14.243%**	**14.246%**	**14.247%**	-	-	-	-
16	Saudi Arabia	**0.598%**	4.724%	3.395%	3.395%	4.029%	4.289%	**2.612%**	**2.612%**	10.167%	**3.939%**	4.547%	4.547%
17	Canada	5.661%	**0.861%**	**0.862%**	**0.863%**	7.801%	**0.674%**	**0.676%**	**0.677%**	10.379%	**0.791%**	**0.793%**	**0.794%**
18	Bangladesh*	5.516%	**2.097%**	**2.099%**	**2.099%**	9.975%	**3.061%**	**3.064%**	**3.065%**	–	–	–	–
19	China	14.044%	**5.492%**	**5.495%**	**5.495%**	14.165%	**8.987%**	**8.976%**	**8.973%**	**14.158%**	16.806%	16.781%	16.776%
20	Qatar	4.045%	**0.780%**	**0.777%**	**0.775%**	5.683%	**2.302%**	**2.293%**	**2.286%**	7.766%	**4.616%**	**4.600%**	**4.589%**

Bold values represent the best scores

The symbol (*) indicates insufficient data in L_15, G_15, R_15 and S_15

^9^).

The rest of the appendix is organized as follows. On the one hand, we present fits to the curves until the selected days, obtaining an MAE for each model and country. On the other hand, the tables of the MAEs made in the predictions are shown. To facilitate the comparison of results, MAEs are normalized by the total number of positive cases on the selected days. From these results, we can conclude that the Gompertz model outperforms in predictive capacity the other PG models and it is recommended to predict new cases of COVID-19.

To abbreviate the headings in the following tables, the Logistic (L), Gompertz (G), Richards (R), and Stannard (S) functions are concatenated with the number of days to be predicted (5, 10, and 15) using the symbol "_". Therefore, column *G_10*, for example, shows the normalized MAEs obtained for the next 10-day forecast, having fitted the data with the Gompertz function.

## Code availability

Custom code programming with Python.
